# Diagnosis of a Strangulated Laparoscopic Incisional Hernia with Point-of-Care Ultrasonography

**DOI:** 10.5811/westjem.2015.3.25498

**Published:** 2015-04-09

**Authors:** Niran Argintaru, Ahmed Al-Den, Jordan Chenkin

**Affiliations:** * University of Toronto, Division of Emergency Medicine, Toronto, Ontario, Canada; † Sunnybrook Health Sciences Centre, Toronto, Canada

## Abstract

The use of point-of-care ultrasound for the diagnosis of bowel obstructions and hernias is becoming increasingly common in the emergency department (ED). Using a relatively rare case of an incisional port hernia, we demonstrate the ultrasound findings of a strangulated hernia causing a partial small bowel obstruction. A 46-year-old female presented four days following a laparoscopic surgery complaining of abdominal pain, nausea and lack of bowel movements. There was a palpable mass in the left lower quadrant under the 12mm trocar port incision. ED point-of-care ultrasound revealed herniated akinetic loops of bowel through her laparoscopy incision. This is the first case report to describe the use of point-of-care ultrasound for the diagnosis of a strangulated incisional port hernia at the bedside.

## INTRODUCTION

Incisional hernias are a well described surgical complication and a common emergency department (ED) presentation. Despite the predominance of laparoscopic surgery, port hernias remain a rare complication with an incidence as low as 0.14% to 6%.^1^,^2^ They are most commonly associated with trocars with a diameter greater than 10 mm.^1^,^2^ Any hernia, including a port hernia, may become incarcerated or strangulated, resulting in bowel necrosis and small bowel obstruction (SBO) and necessitating urgent surgical intervention.

The role of point-of-care ultrasonography (POCUS) in the ED management of abdominal pain, including hernias and small bowel obstruction is growing. Multiple studies have demonstrated sensitivities of 93.9–97.7% and specificities of 81.4–92.7% for the diagnosis of SBO using POCUS in the ED setting.^3^–^5^ There are also several case reports in the literature describing the examination of an abdominal hernia with POCUS, including describing ultrasound-guided ED hernia reduction.^6^–^8^ However, we could find no published reports on the use of POCUS for diagnosing strangulated hernias. Here we describe the findings of POCUS in this case of a rare strangulated incisional port hernia and its potential application for the ED diagnosis of all strangulated abdominal hernias.

## CASE REPORT

A 46-year-old woman presented to the ED with left lower quadrant pain associated with nausea and anorexia worsening over the last 48 hours. The pain was constant and progressive and worsened with movement. Four days earlier the patient had undergone a laparoscopic Burch colposuspension for stress incontinence and pelvic organ prolapse. The surgical notes were unremarkable and her post-operative course was uncomplicated. She was discharged home on post-operative day one.

On arrival, she was tachycardic at 110 beats per minute and normotensive at 117/75. She was afebrile, had a normal respiratory rate, but was quite pale and had considerable difficulty transferring from the chair to the examination table. Her abdomen was generally soft, but a firm, focally tender mass was noted in the left lower quadrant. It was unclear based on the physical examination whether this mass was a seroma, hematoma, or hernia. The patient reported she was passing gas but not stool.

On bedside ultrasonography by the emergency physician, dilated fluid-filled loops of bowel were visible in the mass, herniating through a 12mm port site directly under the skin (Figure and Video). Free fluid was noted between the loops of bowel within the hernia sac. The bowel within the mass was entirely akinetic; however, flow could be identified using colour Doppler.

Based on these results, the general surgery team was called and the decision was made to take the patient to the operating room. An interval computed tomography (CT) confirmed a small bowel obstruction due to a laparoscopic port hernia with signs of early ischemic changes secondary to strangulation of small bowel. The patient underwent an urgent laparotomy that identified purple, yet still viable, strangulated small bowel loop without full thickness necrosis. The bowel was reduced and no bowel resection was necessary.

## DISCUSSION

Laparoscopic port incisional hernias are uncommon, and can be difficult to distinguish from benign fluid collections such as post-operative hematomas or seromas. While most hernias are easily palpable on exam, some cases of Spigelian hernias may not be palpable and ultrasonography has been an established tool in their diagnosis.^8^ In this case, POCUS allowed us to quickly differentiate between a benign postoperative fluid collection and a strangulated hernia.

To examine for a hernia using POCUS, a high-frequency linear transducer is placed over the region of swelling or pain. For evaluation of deeper hernias, a low-frequency curvilinear transducer should be used. The region should be evaluated systematically in two orthogonal planes. On ultrasound, hernias appear as loops of bowel trapped within an echogenic sac protruding through a defect in the abdominal wall.

POCUS can be used to examine for signs of bowel obstruction and strangulation within the hernia sac. A study comparing abdominal radiographs to POCUS for bowel obstruction found that abdominal radiographs had a sensitivity of 46.2% and a specificity of 66.7% when diagnostic, but were non-diagnostic 36% of the time. POCUS on the other hand was found to be 91% sensitive and 84% specific, with no non-diagnostic scans.^3^ Using POCUS, small bowel obstruction should be suspected when there are dilated fluid-filled loops of bowel (>25mm), to-and-fro movement of bowel contents, and free fluid between the loops of bowel. The main findings of strangulation on POCUS include an edematous bowel wall (wall thickness >3mm) with echogenic fat, loss of peristalsis and fluid within the hernia sac.^9^ Late presenting strangulation can have reduced or absent colour flow on colour Doppler and may require bowel resection.^9^–^11^ It is important to note that since absent colour Doppler flow is a late finding of bowel strangulation, Doppler ultrasound is not a sensitive modality for the diagnosis of bowel strangulation. As venous and lymphatic vessel walls are thin, they are readily compressible, resulting in a loss of venous flow significantly earlier than loss of arterial flow.^10^ Detecting venous flow on ultrasound is difficult and is rarely attempted at the bedside.

## CONCLUSION

Point-of-care ultrasound has been shown to be an effective tool for the diagnosis of bowel obstruction and hernias. In this case, timely access to emergency POCUS allowed us to quickly identify a strangulated incisional port hernia. Presence of Doppler flow does not rule out strangulation, while absence of Doppler flow is a late finding. Further studies are needed to evaluate the accuracy of POCUS findings for strangulated hernias.

## Figures and Tables

**Figure:**
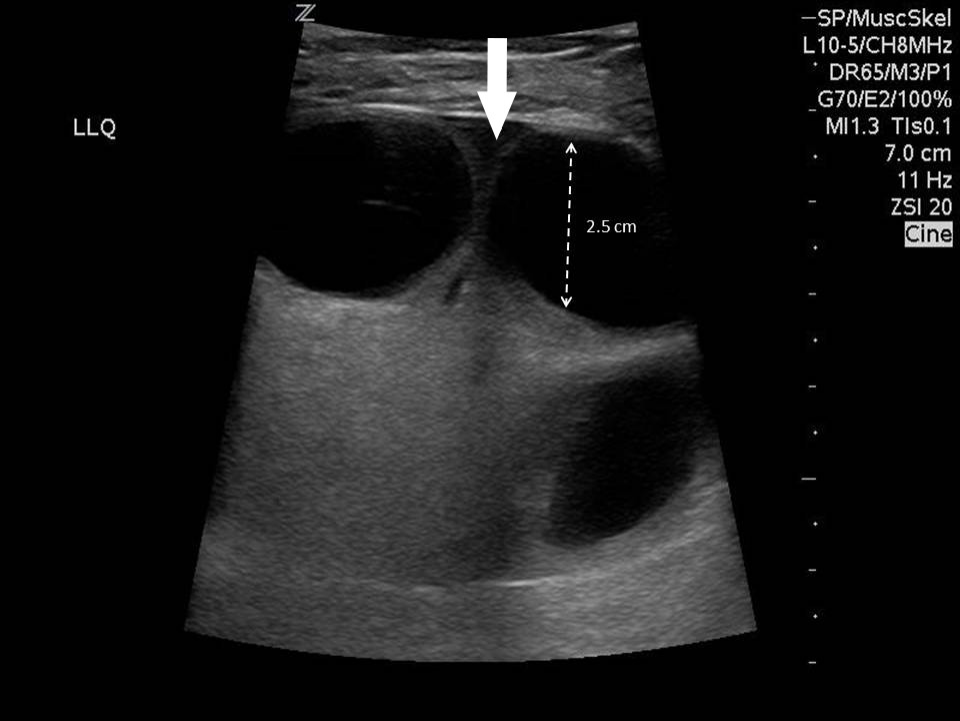
Dilated fluid-filled loops of bowel visible within a herniating mass. Fluid is seen between bowel loops (arrow).

**Video:** A narrated recording of a POCUS scan of a strangulated incisional hernia. Note the dilated akinetic loops of bowel, free fluid between the bowel loops and edematous bowel wall.
